# Expert opinion document: “Electrical impedance tomography: applications from the intensive care unit and beyond”

**DOI:** 10.1186/s44158-022-00055-6

**Published:** 2022-06-21

**Authors:** Michela Rauseo, Elena Spinelli, Nicolò Sella, Douglas Slobod, Savino Spadaro, Federico Longhini, Antonino Giarratano, Cinnella Gilda, Tommaso Mauri, Paolo Navalesi, SIAARTI Study Group

**Affiliations:** 1 Department of Anesthesia and Intensive Care Medicine, University of Foggia, Policlinico Riuniti di Foggia, Foggia, Italy; 2 Department of Anesthesia, Critical Care and Emergency, Fondazione Istituto di Ricovero e Cura a Carattere Scientifico Ca’ Granda Ospedale Maggiore Policlinico Milan, Milano, Italy; 3 Instiute of Anesthesia and Intensive Care, Padua University Hospital, Padova, Italy; 4 Department of Critical Care Medicine, McGill University, Montreal, Quebec, Canada; 5 Anesthesia and Intensive Care Unit, Department of Translational Medicine, University of Ferrara, Ferrara, Italy; 6 Anesthesia and Intensive Care Unit, Department of Medical and Surgical Sciences, “Magna Graecia” University, “Mater Domini” University Hospital, Catanzaro, Italy; 7 Department of Surgical, Oncological and Oral Science (Di.Chir.On.S.), Section of Anaesthesia, Analgesia, Intensive Care and Emergency, Policlinico Paolo Giaccone, University of Palermo, Palermo, Italy; 8 Department of Pathophysiology and Transplantation, University of Milan, Milan, Italy; 9 Department of Medicine – DIMED, University of Padua, Padova, Italy

**Keywords:** EIT, Respiratory monitoring, PEEP, Recruitment maneuvers, Non-invasive ventilation, NIV, CPAP, COVID-19, Intensive care unit, VILI, P-SILI, PEEP, Operating room, Protective ventilation, Mechanical ventilation, ARDS, COPD, ARF, HFNC

## Abstract

Mechanical ventilation is a life-saving technology, but it can also inadvertently induce lung injury and increase morbidity and mortality. Currently, there is no easy method of assessing the impact that ventilator settings have on the degree of lung inssflation. Computed tomography (CT), the gold standard for visually monitoring lung function, can provide detailed regional information of the lung. Unfortunately, it necessitates moving critically ill patients to a special diagnostic room and involves exposure to radiation. A technique introduced in the 1980s, electrical impedance tomography (EIT) can non-invasively provide similar monitoring of lung function. However, while CT provides information on the air content, EIT monitors ventilation-related changes of lung volume and changes of end expiratory lung volume (EELV). Over the past several decades, EIT has moved from the research lab to commercially available devices that are used at the bedside. Being complementary to well-established radiological techniques and conventional pulmonary monitoring, EIT can be used to continuously visualize the lung function at the bedside and to instantly assess the effects of therapeutic maneuvers on regional ventilation distribution. EIT provides a means of visualizing the regional distribution of ventilation and changes of lung volume. This ability is particularly useful when therapy changes are intended to achieve a more homogenous gas distribution in mechanically ventilated patients. Besides the unique information provided by EIT, its convenience and safety contribute to the increasing perception expressed by various authors that EIT has the potential to be used as a valuable tool for optimizing PEEP and other ventilator settings, either in the operative room and in the intensive care unit. The effects of various therapeutic interventions and applications on ventilation distribution have already been assessed with the help of EIT, and this document gives an overview of the literature that has been published in this context.

## Introduction

Electrical impedance tomography (EIT) is a non-invasive tool that displays regional changes in lung volume and the distribution of ventilation at the bedside in real-time, providing information about gas distribution, regional ventilation delay, and, more recently pulmonary perfusion. This dynamic assessment can help clinicians optimize and individualize ventilator parameters tailored to a patient’s characteristics. Indeed, EIT may assist in optimizing mechanical ventilation settings, taking into account the heterogeneity of the lung. In addition, real-time monitoring of lung function can allow clinicians to more accurately predict patient recovery, thereby reducing dependence on the ventilator, and at the same time, avoiding the risks of premature weaning. Being a noninvasive and safe technique, the popularity of EIT is increasing among phyisicians caring for mechanically ventilated patients. This review article provides an overview of the EIT literature with a focus on its application in various clinical scenarios.

### Acute respiratory distress syndrome

In the 1980s, preliminary reports discussed how impedance changes measured within the thorax during spontaneous breathing with an EIT system were linearly correlated with tidal volume [1]. As it became clear that the acute respiratory distress syndrome (ARDS) was characterized by a heterogeneous distribution of ventilation [2], EIT emerged as a practical tool to monitor regional ventilatory abnormalities and guide the titration of mechanical ventilation in patients with ARDS [3–5].

#### Assess recruitability

Recruitability refers to the potential to stabilize the re-opening of atelectatic lung regions with the application of higher positive end-expiratory pressure (PEEP). The observation that ARDS patients present highly variable degrees of recruitability [6] has fueled interest in measuring recruitability at the bedside and suggests that this should be done prior to selecting a personalized PEEP. Increasing PEEP in the absence of recruitability is associated with deleterious consequences including hemodynamic depression and overdistension. A simple bedside maneuver based on respiratory mechanics can estimate recruitability with higher PEEP [7], but compared to EIT-based methods, this technique cannot be used during spontaneous breathing or for continuous monitoring and only provides a global assessment.

The change in end-expiratory lung impedance measured by EIT allows for the calculation of the volume recruited by changing PEEP [8]. This volume is then divided by the change in PEEP to obtain its compliance. Finally, compliance of the recruited lung is divided by the compliance of the respiratory system at lower PEEP (i.e., the size of the baby lung) to obtain the recruitment to inflation (R/I) ratio. Values above 0.5–0.7 indicate higher recruitability. The regional R/I ratio of the dorsal lung may be even more sensitive for the estimation of recruitability.

Importantly, EIT assesses recruitability at a regional level and provides crucial information about whether recruitment in the dorsal lung has occurred at the cost of concomitant overdistension in the ventral areas. EIT has shown heterogeneity in regional lower inflection points (LIP) and upper inflection points (UIP) of pressure-volume curves in ARDS patients [9, 10], with higher regional LIP indicating need for higher PEEP to restore aeration and airway patency.

Finally, improved compliance of the most dorsal regions at higher PEEP may indicate recruitability in a dynamic and simple fashion [11].

#### Setting positive end-expiratory pressure

In 1975, Suter et al. proposed that the optimal PEEP resulted in the highest respiratory system compliance, the lowest dead-space fraction, and the greatest oxygen delivery [12]. In the era of lung protective ventilation, the benefits of improved oxygen transport must be balanced with the risks of local alveolar overdistension and ventilator-induced lung injury [13].

Costa et al. described an EIT-based approach to assess collapse and overdistension by comparing EIT to CT scanning [14]. By assessing the tidal change in lung impedance divided by the driving pressure they proposed a “per pixel” compliance. The relative changes in pixel compliance during a decremental PEEP trial allowed quantification of the regional effects of PEEP on lung mechanics. Loss of compliance associated with increasing PEEP was termed “overdistension” whereas loss of compliance associated with decreasing PEEP was termed “collapse.” Knowing the percentage of overdistended and collapsed pixels at each level, optimal PEEP was defined as the value that maximized recruitment and minimized overdistension. This approach often provides an incentive to reduce PEEP [15].

Once PEEP is set, EIT can ensure that recruitment is maintained. Eronia et al. [16] performed a PEEP titration targeting stability of end-expiratory lung volume. This method defined a PEEP that maintained lung recruitment in most patients and led to the selection of higher PEEP compared to a standard PEEP to FiO_2_ table. This approach was also associated with an improvement in respiratory system compliance and oxygenation.

An important caveat to EIT is that it provides measurements of lung volume that are based on relative values between two states. Furthermore, the range of start and end PEEP values included in the decremental trial can potentially mislead the clinician if too narrow a range is used and impact the EIT-derived optimal PEEP. The following case study show the an integrated approach used to identify an appropriate setting of ventilation.

#### Case study

**Fig. 1:**
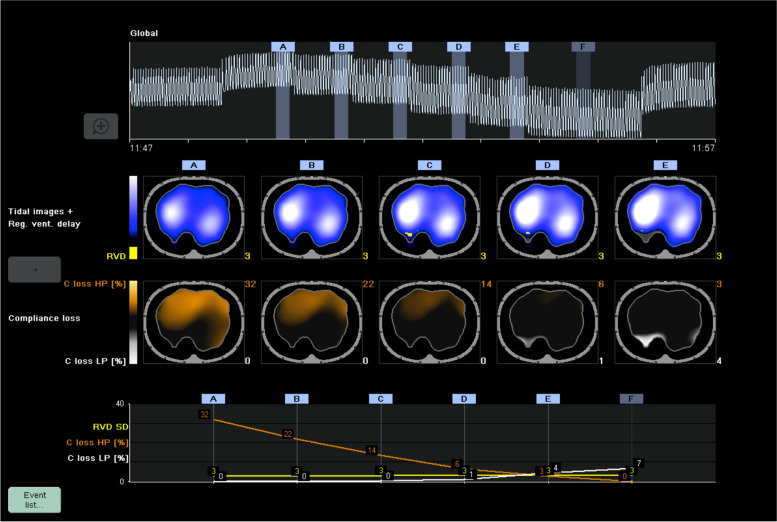
demonstrates a regional analysis of lung mechanics obtained during the decremental PEEP trial, provided as a diagnostic tool. Alveolar overdistension is represented in orange as a “compliance loss” (C loss HP) that occurred at higher PEEP (more overdistension at time point A (PEEP 18 cmH_2_O) compared to B (PEEP 16 cmH_2_O)). Alveolar collapse is represented in white as a “compliance loss” (C loss LP) that occurred at lower PEEP (more collapse at time point F (PEEP 8 cmH_2_O) compared to E (PEEP 10 cmH_2_O)). The crossover point representing minimal overdistension and atelectasis was 10 cmH_2_O (time point E). Below this value, dorsal collapse increased. Above this value, no collapse was detected, but increasing ventral overdistension developed. This example illustrates how EIT might be used as an incentive to reduce PEEP and optimize regional lung mechanics in a patient with ARDS

A 35-year-old man was admitted to the ICU with a diagnosis of ARDS secondary to COVID-19. He was sedated and paralyzed while receiving controlled mechanical ventilation with a volume-controlled mode. The baseline total PEEP was 14 cmH2O. Airway driving pressure was 14 cmH2O and exhaled VT was 429 mL with a RR of 2 23 and FiO2 of 0.4. The R/I ratio was calculated as 0.44, suggesting a lower potential for lung recruitment at higher PEEP. A decremental PEEP trial was performed to personalize this patient’s PEEP. The ventilator mode was switched to pressure-control (PC) with a driving pressure (PC above PEEP) set at 15 cmH2O throughout the trial. First, PEEP was increased to 18 cmH2O (time point A on Fig. 1) and maintained for 1 minute. Then PEEP was decreased by increments of 2 cmH2O until a PEEP of 8 cmH2O was achieved, with each PEEP level maintained for 1 minute (time points B through F). Respiratory mechanics and oxygen saturation recorded during each step reported in Table 1.

**Table 1:** Respiratory mechanics and oxygen saturation recorded during each step

	Baseline PEEP 14 cmH_2_O	PEEP 18 cmH_2_O	PEEP 16 cmH_2_O	PEEP 14 cmH_2_O	PEEP 12 cmH_2_O	PEEP 10 cmH_2_O	PEEP 8 cmH_2_O
EIT image reference letter		A	B	C	D	E	F
Ventilator mode	VCV	PCV	PCV	PCV	PCV	PCV	PCV
VT_E_ (mL)	429	390	446	511	558	553	587
Driving pressure (cmH_2_O)	14	15	15	15	15	15	15
C_RS_ (mL/cmH_2_O)	30.6	26	29.7	34.1	37.2	36.9	39.1
SpO_2_	91	93	94	95	96	95	95

#### Non-intubated patients

There is increasing interest in preserving spontaneous breathing in patients with ARDS and understanding whether such an approach is lung protective [17]. In this setting, EIT can assist in titrating respiratory support and identifying the consequences of excessive respiratory drive and effort that increase regional lung stress and strain [18, 19].

Yoshida et al. used EIT to demonstrate that a switch to spontaneous breathing in an ARDS patient could result in a nearly twofold increase in ventilation distributed to dependent lung zones [20]. This was accompanied by deflation of the ventral lung shortly after initiation of the breath. This movement of air from one region of the lung to another, termed “occult pendelluft”, can cause significant local overdistension.

In ARDS patients treated with pressure support ventilation, Mauri et al. used EIT to demonstrate a more homogenous distribution of ventilation when the pressure support level was titrated to a physiologic range of P_0.1_ [21]. EIT can also be used to assess the clinical impact of respiratory support with high flow nasal cannula [22] and identify patients at risk for failure during support with non-invasive ventilation [23].

### Acute exacerbations of COPD

Chronic obstructive pulmonary disease (COPD) is characterized by a heterogenous increase of regional airway resistances and lung compliances. In stable patients with COPD, this pathologic increase in regional time constants can be visualised by EIT as a heterogeneous distribution of ventilation and variable filling/emptying times across lung units [24, 25]. Acute exacerbations of COPD (AECOPD) are a frequent cause of presentation to the emergency department and clinicians face the difficult challenge of balancing effective CO_2_ washout with the risk of dynamic hyperinflation when providing respiratory support. EIT can aid in monitoring and managing these patients, particularly in titrating ventilatory support and assessing response to therapy (Table 2).

**Table 2:** Pathophysiology of COPD as assessed by EIT

**Spatial lung heterogeneity**	
**Pathophysiological variable**	**EIT parameter**
Regional dynamic hyperinflation/intrinsic PEEP [26].	Regional decrease of end-expiratory lung volume during decremental PEEP trial [27]. Regional end-expiratory flow not returning to zero before next breath [28].
Heterogenous distribution of ventilation within the lungs.	Elevated global inhomogeneity index [29]. Uneven distribution of ventral/dorsal ventilation [30].
**Temporal lung heterogeneity**	
**Pathophysiological variable**	**EIT parameter**
Heterogeneity of regional start of inflation/deflation—regional differences in time constants.	Out of phase filling and emptying of different regions of the lung [25, 31]. Regional ventilation-delay index [32].
Regional pendelluft [33].	Volume shifts between lung regions during end inspiratory hold. Time difference between global and regional impedance versus time curves [24, 33].

In ventilated patients with AECOPD, dynamic hyperinflation (intrinsic PEEP) can occur due to the increased time constants of lung units and shorter expiratory time due to a higher respiratory rate. If the externally applied PEEP is inadequate, intrinsic PEEP results in increased work of breathing and an inhomogeneous distribution of ventilation. In this setting, EIT can be used to set an optimal PEEP that minimizes the consequences of dynamic hyperinflation. Interestingly, Kostakou et al. performed a PEEP titration assessing ventilation heterogeneity using EIT in a mechanically ventilated patient with a severe AECOPD and evidence of dynamic hyperinflation [26]. They set PEEP to 0%, 50%, 80%, 100%, and 150% of the globally measured intrinsic PEEP and measured the regional delay of ventilation, the time for a lung region to attain a certain impedance change [32]. An optimal homogeneity with the lowest delay of ventilation was achieved at a PEEP set to 80% of the intrinsic PEEP. Interestingly, this PEEP value also resulted in the greatest exhaled tidal volume, but not the greatest respiratory system compliance.

In an intubated patient with COPD, Mauri et al. demonstrated the usefulness of EIT in selecting a personalized external PEEP in the setting of intrinsic PEEP [27]. During a decremental PEEP trial, EIT displayed that the PEEP level at which dependent lung regions stopped deflating (indicative of the quantity of regional intrinsic PEEP) was higher than for non-dependent. PEEP was set at a level corresponding to the highest level of regional intrinsic PEEP, and the patient was successfully transitioned to assisted ventilation. Importantly, this PEEP level was higher than the traditionally measured global value obtained during an end-expiratory occlusion.

Karagiannidis et al. demonstrated the feasibility and reliability of measuring regional time constants using EIT [31]. They reported significant heterogeneity and overall increased time constants in invasively ventilated patients with AECOPD compared to ARDS. Moreover, they detected regional differences in airflow limitation and the response to different levels of applied PEEP.

The heterogeneity of time constants in patients with COPD can cause an asynchronous pattern of ventilation giving rise to occult pendelluft and regional overdistension. In patients with AECOPD, Sang et al. demonstrated significant heterogeneity in the magnitude and timing of impedance versus time curves in different regions of the lung [33]. These “phase shifts” and the heterogeneity of amplitude differences indicated delays between emptying of different lung units. The magnitude of EIT measured expiratory delays worsened with increasing airway resistance and improved after administration of bronchodilator therapy, suggesting that EIT can be a helpful adjunct in monitoring patients with AECOPD over time.

By measuring flow versus time curves at end-expiration, Zhao et al. also used EIT to identify regional air-trapping and assess the response to bronchodilator therapy in patients with AECOPD [28].

In terms of ventilation mode, in patients with AECOPD receiving assisted (pressure support) ventilation, Sun et al. used EIT to demonstrate that switching to a neurally adjusted ventilatory assist mode increased the homogeneity of the distribution of ventilation and reduced the work due to trigger [30].

Altogether, a nuanced approach to ventilator management and a PEEP selection that optimizes work of breathing in patients with AECOPD may be facilitated with an EIT-guided approach.

### COVID-19 acute respiratory failure

The novel coronavirus disease 2019 (COVID-19) pandemic has led to an overwhelming amount of mechanically ventilated patients [34] with severe hypoxemic acute respiratory failure (hARF) consequent to either alveolar or vascular injury or both [35]. EIT has been proposed as a valuable tool to personalize the management of COVID-19 patients with hARF [36–40].

Recent data indicate that limiting driving pressure (DP) as much as possible reduces the risk of death in mechanically ventilated COVID-19 hARF patients [41]. By estimating the loss of compliance due to lung collapse and overdistension [14], EIT offers the possibility to minimize DP by individualizing PEEP selection [42].

Sella et al. [38], in a cohort of intubated COVID-19 patients, found that the median PEEP selected by EIT (PEEP_EIT_) that minimized the overall loss of compliance was 12 cmH_2_O [interquartile range 10–14 cmH_2_O] [43] and corresponded to the intersection between the EIT alveolar collapse and overdistension curves [14]. Notably, the loss of lung compliance due to lung collapse observed with PEEP values from the lower PEEP/FiO_2_ table was comparable to PEEP_EIT_, whereas the loss of lung compliance due to lung overdistension was significantly greater with PEEP values from the higher PEEP/FiO_2_ table than with PEEP_EIT_, suggesting better agreement between PEEP_EIT_ and the lower PEEP/FiO_2_ table [38]. In keeping with these results, Perier et al., in a series of 17 COVID-19 hARF patients, found a median PEEP_EIT_ of 12 [9-12] cmH_2_O, without significant differences between patients with respiratory system compliance (Crs) ≥ 40 mL/cmH_2_O and <40 mL/cmH_2_O [39].

In contrast, Van der Zee et al. found higher values of PEEP_EIT_ (21 [16–22] cmH_2_O), closer to those advised by the higher PEEP/FiO_2_ table [40]. These discrepancies may be partly explained by the different criteria used for PEEP_EIT_ selection in this study, set 2 cmH_2_O above the intersection of the curves representing the cumulative percentage of compliance loss due to lung collapse and overdistension [40]. Furthermore, Van der Zee et al. enrolled more obese patients (median body mass index of 30.0 [27.0–34.0] kg/m^2^ [40], compared to Sella et al. (26.2 [25.4–30.9] kg/m^2^) [38], perhaps explaining the higher PEEP in the setting of reduced chest wall compliance [44].

A scientific dispute among opinion leaders has debated the use of noninvasive respiratory supports in COVID-19 hARF. While some authors are concerned about the risk of patient self-inflicted lung injury [45], others are cautious, considering the harms of unnecessary intubation [46]. Indeed, duration of NIV use [47, 48] and location of application [48] have been associated with hospital mortality in COVID-19 patients intubated after NIV failure. EIT has been proposed as a tool to assess the response to continuous positive airway pressure (CPAP) and recognize patients at risk for CPAP failure [23]. In a series of 10 patients admitted to the ICU for COVID-19 pneumonia and supported with CPAP, Rauseo et al. performed an EIT-guided decremental PEEP trial from 12 cmH_2_O to 6 cmH_2_O and found that a reduction of EELI smaller than 40% after PEEP de-escalation predicted CPAP failure [23].

COVID-19 hARF is characterized not only by alveolar injury, but also by severe pulmonary vascular disruption [49] with small- and mid-sized pulmonary vessel thrombosis [35], associated with a hypercoagulable state [50, 51]. Recent data from COVID-19 patients suggest the potential of an EIT perfusion assessment to detect both ventilation-perfusion (V/Q) mismatch [52–54] and pulmonary vasculature alterations, consistent with findings of computed tomography pulmonary angiography [55, 56].

Prone positioning has been widely applied in COVID-19 hARF, with 61% of intubated patients undergoing at least one cycle of prone positioning [56].

Nevertheless, the mechanisms underlying the improvement in oxygenation after prone positioning in COVID-19 patients remain unclear.

Zarantonello et al. described the case of one COVID-19 hARF patient, studied with EIT ventilation-perfusion analysis in the supine position and 60 min after being turned prone, and found that prone positioning increased ventilation in the dorsal areas and shifted perfusion to the ventral areas, overall improving V/Q matching [52]. Perier et al., in 17 COVID-19 hARF patients [39], found no difference between PEEP_EIT_ in the supine and prone position and no improvement in DP and Crs after turning patients prone, thereby casting doubt on the role of alveolar recruitment in the improvement of arterial oxygenation during prone positioning. Subsequently, Perier et al., in a cohort of 9 patients with COVID-19 hARF, showed that turning patients from the supine to prone position decreased ventral dead space and dorsal shunt with a trend towards an improvement in V/Q matching, especially in the ventral areas of the lung [53].

### Anesthesia and perioperative period

EIT monitoring during the perioperative period can improve the care of patients undergoing different surgical procedures and aims to reduce post-operative pulmonary complications by identifying factors that may benefit from a personalized ventilator strategy.

#### Anesthesia induction

A certainty of anesthesia induction is a decrease in functional residual capacity, the magnitude of which is unpredictable (FRC) [57, 58]. Ideal ventilator management aims to maintain end-expiratory lung volume at a value that is as similar as possible to preoperative FRC. Reductions in FRC result in derangements of the blood gases and ventilation/perfusion mismatch [59–61].

On the other hand, an unphysiological increase in FRC should be considered unsafe, as this may augment lung stress [62]. Difficulty in providing an optimal ventilator strategy is probably due to the limited value of information available during perioperative ventilation, such as plateau pressure or tidal volume per kilogram of body weight, which can only weakly characterize the mechanical properties of the respiratory system [63]. Furthermore, with the adoption of “protective ventilation” in the operative room, the modern concept of driving pressure finds its application, only a very low external PEEP is given, for reliable information about dynamic strain [64]. Based on the recent literature, EIT monitoring may play a pivotal role in providing a bedside assessment of FRC.

**Fig. 2:**
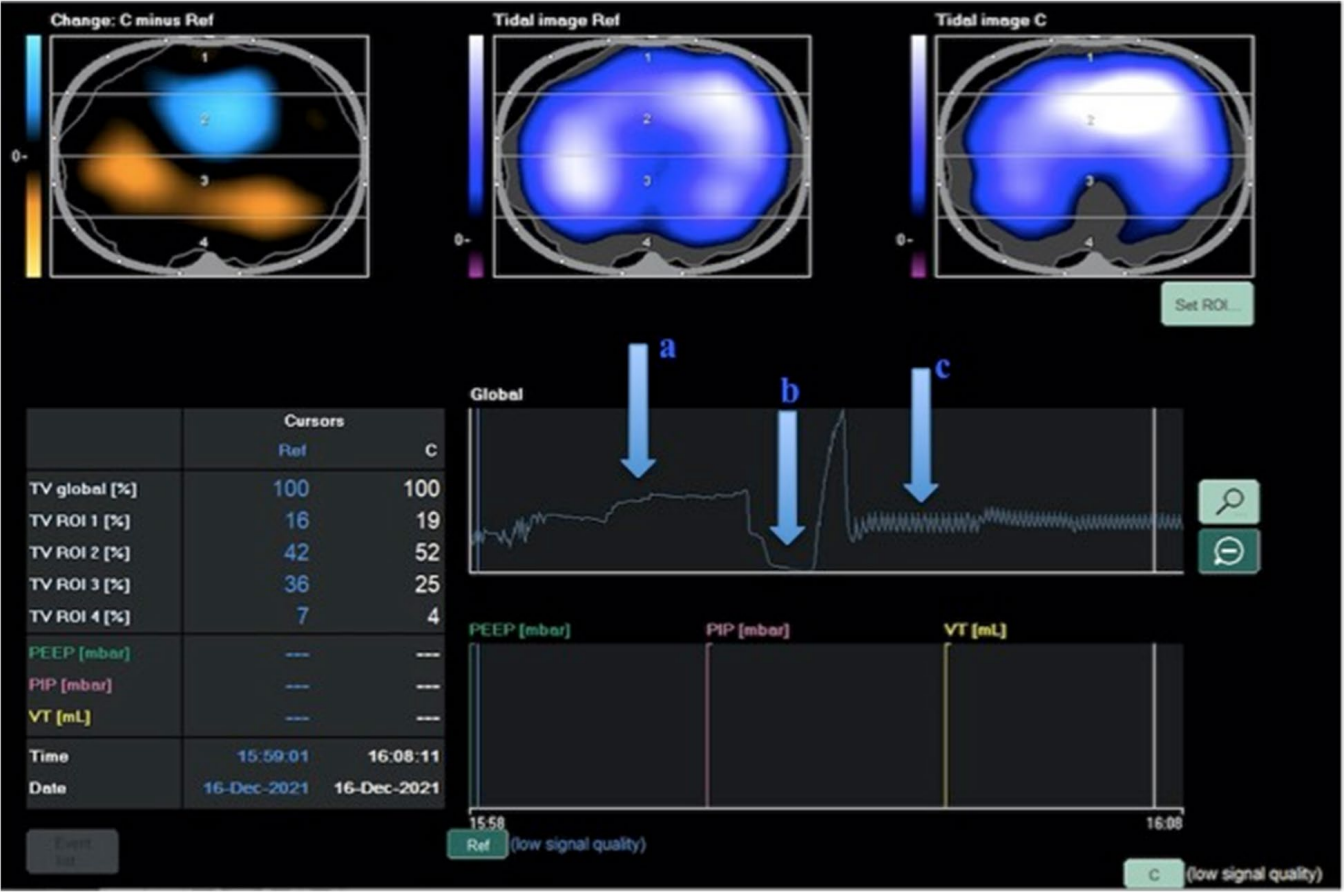
From spontaneous breathing (**a**) to induction of anesthesia and ventilation in bag mask (**b**) and controlled mechanical ventilation, followed by the application of an external PEEP (**c**)

EIT demonstrated a decrease in FRC after anesthesia induction that was reversed by the application of PEEP in morbidly obese patients undergoing laparoscopic gastric bypass surgery [65]. In these patients, pre-oxygenation with a tight-fitting mask and 10 cmH_2_O of PEEP transiently increased FRC and prevented hypoxemia during anesthesia induction. Nestler et al. demonstrated that bag-mask ventilation without applied PEEP resulted in a significant decrease in post-intubation FRC [66]. Therefore, EIT represents an encouraging opportunity to monitor the efficacy of different pre-oxygenation strategies (Fig. 2).

#### Intraoperative mechanical ventilation

As mentioned above, the loss of FRC during general anesthesia is unpredictable, due to many contributing factors including a patient’s pathophysiology, anesthesia technique, body positioning, and/or surgical procedures. It is, however, well known that this loss of FRC results in atelectasis in more than 90% of patients [57–59]. Ukere et al. [67, 68], using EIT in both anesthetized and awake patients identified under ventilated “silent spaces,” in different body positions. The availability of this information at the bedside may guide the setting of external PEEP, limiting the development of atelectasis and consequences related to PPC and infections. During general anesthesia, based on a breath-by-breath analysis, electrical impedance tomography can highlight changes in lung aeration and the distribution of ventilation, thereby allowing the clinician to titrate mechanical ventilation on the basis of a patient’s regional respiratory mechanics [69]. In the last few years, research based on optimizing PEEP guided by EIT at the bedside identified a method for determining the “best” PEEP as that which minimizes alveolar overdistension and collapse, limits driving pressure and optimizes oxygenation [70]. Indeed, Pereira et al. showed that the effect of this EIT-based approach was more considerable during laparoscopic procedures compared to laparotomies. Nestler et al. [66] proposed another EIT-derived parameter to set PEEP in obese patients undergoing general anesthesia: the regional ventilation delay index (RVDI), defined as the standard deviation of Regional Ventilation Delay (a measure of the temporal delay in ventilation of regions of the lung) in all pixels. A lower RVDI indicates a more homogeneous distribution of ventilation and thus may limit derecruitment. Their protocol consisted of a recruitment maneuver, followed by the application of PEEP titrated to minimize RVDI. When compared to a fixed PEEP (5 cmH_2_O), this method led to a significant improvement in oxygenation and better regional homogeneity, with no deleterious postoperative effects.

One of the most useful and fascinating parameters obtained from EIT to evaluate the effects of different PEEP levels is the End Expiratory Lung Impedance (EELI), which represents the impedance at the end of expiration. Changes in EELI reflect lung recruitment due to PEEP. Using this parameter, Erlandsson et al. [65] showed that, in obese patients undergoing laparoscopic surgery, an increase or decrease in the slope of EELI following a change in PEEP, corresponded to recruitment or derecruitment, respectively, whereas a stable end-expiratory lung volume reflected optimal PEEP. Eronia et al. [16], in patients with acute respiratory failure, showed that it is possible to measure recruitment from the variation in EELI measured at the beginning and end of a recruitment maneuver and that PEEP could be titrated according to the change in ∆EELI. This method might even be useful during general anesthesia: an increase in EELI lower than that predicted by the recruited volume could be helpful in suggesting the presence of lung overdistension [71].

EIT application does not exclusively describe how to set ventilation or physiology of the respiratory system. It has also been used during thoracic surgery to confirm the correct positioning of double lumen endotracheal tubes (DLT) and titrate the optimal combination of tidal volume and PEEP in patients requiring one-lung ventilation (OLV). Compared to the gold standard fiberoptic bronchoscopy for routine confirmation of the correct positioning of DLT, EIT has the advantage of allowing clinicians to non-invasively identify any misplacement of DLTs in the contralateral main bronchus by accurately displaying left and right lung ventilation [72]. Transitioning from two-lung ventilation (TLV) to OLV, the mechanical properties of the ventilated lung undergo significant changes. The exclusion of one lung from ventilation in the lateral decubitus position determines a change in lung compliance, resistance, and the distribution of tidal volume. Hence, the ventilatory management of these patients is challenging and tidal volume and PEEP should be frequently reassessed during each surgery step [60, 73]. Each of these physiologic variations can be detected by EIT. Using an index of inhomogeneity derived from EIT (GI, global inhomogeneity index), Zhao et al. [74] showed that it is possible to titrate the combination of PEEP and TV in patients shifting from TLV to OLV. In their study, they found a good degree of inter-patient equivalence and the GI correlated with the gas distribution in the lung. The same authors recently explored if the regional ventilation distribution (measured by EIT) and PaO_2_ could help titrating TV and PEEP during OLV [75].

Future perspectives and large RCTs will elucidate the usefulness of EIT in setting intraoperative ventilation and clarify whether the use of EIT reduces the incidence of postoperative pulmonary complications (PPC).

#### Post-extubation period

**Fig. 3:**
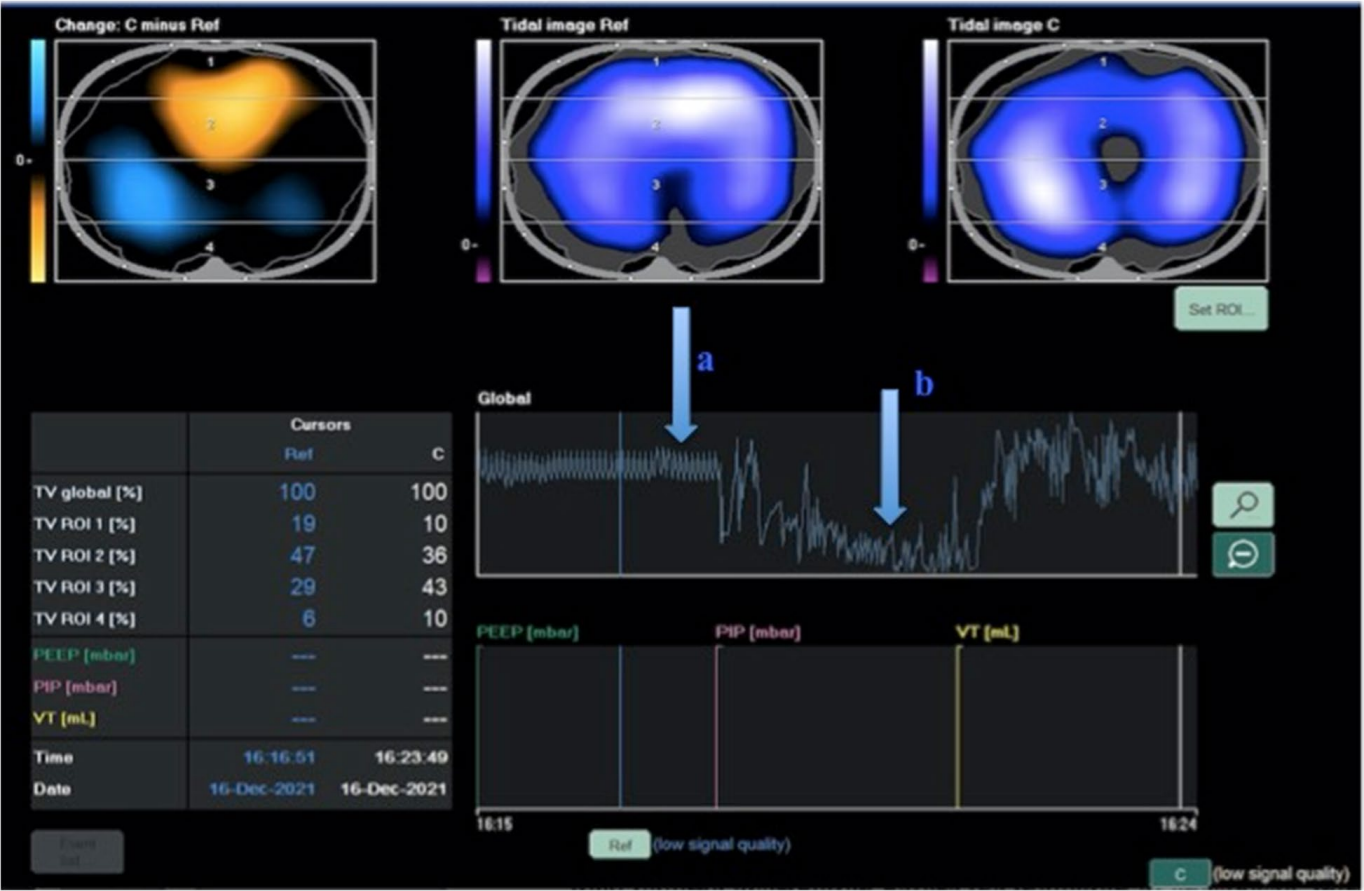
In the first part of the recording, the patient was still intubated and under controlled mechanical ventilation (**a**). After the weaning process, it is possible to follow the extubation period and the patient breathing spontaneneusly (**b**), followed by the application of a postoperative CPAPs

EIT also allows for the continuous monitoring of patients in the postoperative period (Fig. 3). The end of surgery and subsequent post-extubation phase are times that require close monitoring due to the abrupt discontinuation of mechanical ventilation and loss of respiratory monitoring that was provided by the ventilator. In addition, these changes coincide with the persisting consequences of sedation, including muscle weakness, reduced inspiratory effort and transpulmonary pressure, an impaired cough reflex and ability to clear secretions due to residual paralysis, and/or poor pain control. Altogether, these factors contribute to an increased risk of postoperative pulmonary complications, which might easily be identified by monitoring changes in ventilation distribution and EELI measured by EIT, especially at the end of the surgical procedure. Schaefer et al. [69] described the feasibility of using EIT to monitor regional tidal volume distribution before the induction of anesthesia, intra-operatively, after extubation, and in the post-operative period. The authors showed that during general anesthesia, tidal ventilation is distributed to the ventral part of the lungs due to muscle paralysis. When spontaneous breathing is restored and following extubation, ventilation, and re-aeration of the dorsal part of the lungs take place, increasing the homogeneity of ventilation, decreasing the tendency for atelectasis. Interestingly, despite using a personalized intra-operative PEEP setting and a recruitment maneuver before extubation, early post-operative EELV is lower compared to baseline before induction of anesthesia [66]. A decrease in EELV at the end of surgery might represent an “alarm bell” that suggests an increased risk of developing postoperative atelectasis and extubation failure. For this reason, EIT monitoring can help to identify patients with a reduced post-operative EELV who might benefit from post-extubation non-invasive ventilation and early mobilization (i.e., obese patients) [76]. Karsten et al. used EIT to evaluate the impact of low versus high PEEP during laparoscopic surgery on post-operative ventilation distribution and showed that a higher intra-operative PEEP resulted in a more homogeneous distribution of ventilation in the early post-operative period [77].

### Weaning

Weaning is the entire process leading patients to the discontinuation of mechanical ventilation and extubation [78]. A spontaneous breathing trial (SBT) is commonly performed to determine whether weaning has been successful and the patient is ready for extubation. While various clinical parameters are utilized to define SBT success, the most powerful predictor of weaning success is the respiratory rate (RR) to tidal volume (Vt) ratio (RR/Vt) [78]. About one fifth of patients, with rates varying from 14 to 31% among studies, fail their first SBT attempt and require reinstitution of mechanical ventilation [78]. After a successful SBT, a fraction of patients, ranging from 3 to 19% among studies, develop post-extubation respiratory failure requiring re-intubation, a complication associated with significantly increased mortality [78]. Prophylactic application of non-invasive ventilation (NIV) soon after extubation in patients at risk of post-extubation respiratory failure may prevent the need for re-intubation and improve outcomes [79]. Most studies broadly consider at-risk patients to be those older than 65 years old or with underlying cardiac or respiratory disease [79]. It is, therefore, of paramount clinical importance to improve the precision of weaning and extubation failure predictions and recent studies indicate a role of EIT for this purpose.

In a general population of 42 mechanically ventilated patients, Lima et al. assessed the variation of end-expiratory lung impedance (EELI) occurring during a 30-min SBT, as conducted by T-piece (10 patients) or low levels of pressure-support ventilation (PSV) (32 patients). In the T-piece group, irrespective of SBT outcome, EELI progressively declined throughout the SBT, though a significantly greater decrease in EELI was observed in patients failing the SBT [80]. In the PSV group, EELI did not vary significantly during the SBT and no difference in EELI variations was observed between patients with different SBT outcomes, likely because ventilator settings, including PEEP, before and during the SBT were quite similar [80]. No difference in Tidal Impedance Variation (TIV) was observed in both groups [80].

In 78 patients at risk for extubation failure, Longhini et al. applied EIT during an SBT conducted with low (2 cmH_2_O) CPAP applied through the ventilator circuit [81]. The authors also assessed the heterogeneity of air distribution within the lung, using the Global Inhomogeneity index (GI) [81]. Compared to weaning successes, patients failing the SBT were characterized by a greater loss in EELI during the SBT and a greater GI at baseline and during the course of the SBT [81]. Again, no difference in TIV was observed between SBT successes and failures [81].

In 31 patients experiencing prolonged weaning, Bickenbach et al. also reported that T-piece SBT failure was characterized by a greater GI at baseline, while gas exchange and RR/Vt were not different between patients succeeding and failing the SBT [82]. Their results suggest that not attempting a SBT in patients with a baseline GI > 41.5 would avoid 87.5% of all SBT failures [82]. Moon et al. recently found that GI was significantly greater in patients failing a T-piece SBT, in a population of 40 patients either with (*n*=16) or without (*n*=24) diaphragm dysfunction [83]. In keeping with these previous results [81–83], in 53 patients mechanically ventilated for more than 72 h and undergoing their first T-piece SBT, Wang et al. further confirmed that GI prior to SBT helps to predict SBT outcome [84].

In a cohort of 30 patients with prolonged weaning, Zhao et al. described different patterns of ventilation according to weaning outcomes; in patients succeeding, ventilation was redistributed towards the dorsal regions, with a more homogeneous distribution between the anterior and posterior regions when decreasing support levels [85].

During the weaning process, Longhini et al. demonstrated that chest physiotherapy as applied by high-frequency chest wall oscillation (HFCWO) improves lung aeration in patients with copious secretions. Also noteworthy, the association of HFCWO with a recruitment maneuver did not produce any further physiological benefit [86].

Finally, the study by Longhini et al. was the only one that investigated the potential of EIT to predict the need for NIV in the post-extubation period. Among 61 patients who successfully passed a SBT, 22 (36.1%) experienced post-extubation respiratory failure within 48 h. Up to 30 min after extubation, no differences in EELI, TIV, or GI were observed between patients succeeding and failing extubation [81].

#### Patient-ventilator dyssynchrony

In order to avoid the consequences of potentially harmful ventilator asynchronies, EIT monitoring used during assisted spontaneous breathing can facilitate the early recognition of breath stacking and pendelluft [87, 88].

Breath stacking may be caused by reverse triggering or double-triggering and results in consecutive inspiratory cycles delivered by the ventilator during an incomplete exhalation [87]. When breath stacking occurs, EIT can demonstrate potentially harmful end-inspiratory lung volumes and is more sensitive when compared to conventional monitoring, which only indicates a modest increase in V_T_ [89].

Pendelluft describes how gas movement between different pulmonary regions results in an uncontrolled and dangerous alveolar de-inflation and inflation, depicted as intrapulmonary asynchrony. EIT allows for the monitoring of a pendelluft phenomenon, since it can readily display air movement within the lung from nondependent to dependent regions even when V_T_ is unchanged [88]. This phenomenon can be caused by excessive diaphragmatic contractions during strong spontaneous efforts and may increase strain of the dependent lung during early inflation. Conventionally monitored respiratory parameters such as flow, volume, and pressure are unable to demonstrate Pendelluft whereas EIT can [88].

#### Noninvasive ventilation

During non-invasive ventilation, global parameters such as pressure-volume curves or the respiratory system compliance do not reliably illustrate what is actually happening in the lung, especially regarding the regional distribution of the administrated tidal volume, where high tidal volumes may lead to patient self inflicted lung injury (PSILI) [90–92].

A lung protective strategy, either during invasive and noninvasive ventilation, should require real-time monitoring of regional lung ventilation to determine the distribution of lung ventilation such as hyperventilation. During the height of the COVID-19 pandemic, Rauseo et al. [23] demonstrated that the number of patients with respiratory failure far exceeded the availability of intensive care unit beds, often prompting physicians to choose non-invasive ventilation as initial therapy. Under such conditions, EIT was identified as a helpful tool to assess the response of patients to NIV and rapidly identify an optimal ventilatory strategy.

Bordes et al. [93] assessed functional residual capacity and ventilation distribution in eighteen spontaneously breathing adult patients undergoing digestive endoscopic procedures under anesthesia and showed that, in awake patients, tidal volume was primarily distributed to the dependent lung (57.5 vs 43.1%; *P* = .009), whereas after anesthesia induction, ventilation shifted to the nondependent lung (43.1% before anesthesia, 58.9% after anesthesia; *P* = .002) with a marked decrease in end-expiratory lung impedance. In the same cohort, application of noninvasive ventilation resulted in a significant increase in end-expiratory lung impedance (*P* = .005) without changing the distribution of ventilation.

Lastly, high flow nasal cannula (HFNC) under EIT monitoring has shown improved oxygenation by increasing both end-expiratory lung volume and tidal volume, regardless of body position suggesting an increase in functional residual capacity [94, 95].

Given the high number of patients treated with NIV/HFNC, large randomized controlled trials are needed; future applications of EIT monitoring could be in thoracic trauma patients and/or pre- and postoperative patients treated with prophylactic NIV/HFNC (to avoid intubation and or complications related to intubation).

## Conclusions

Evidence-based medicine has demonstrated that “one size doesn’t fit all.” Lung monitoring and mechanical ventilation have been enhanced by the use of the EIT system, and despite its limitations, this device represents a remarkable technological advance in the fields of anesthesia and critical care medicine.

### Future perspectives

Future areas of inquiry include using EIT to guide the use of more novel technologies such as helmet NIV or extra-corporeal CO_2_ removal in patients with AECOPD.

Beyond ventilation, a novel feature of EIT is the ability to obtain the distribution of pulmonary blood flow [96, 97] and a pixel-based mapping of ventilation to perfusion ratios. This technology represents an exciting future potential to guide the treatment of patients with ARDS.

## Data Availability

Not applicable.
