# Correction: Expert opinion document: “Electrical impedance tomography: applications from the intensive care unit and beyond”

**DOI:** 10.1186/s44158-022-00059-2

**Published:** 2022-06-30

**Authors:** Michela Rauseo, Elena Spinelli, Nicolò Sella, Douglas Slobod, Savino Spadaro, Federico Longhini, Antonino Giarratano, Gilda Cinnella, Tommaso Mauri, Paolo Navalesi, SIAARTI Study Group

**Affiliations:** 1 Department of Anesthesia and Intensive Care Medicine, University of Foggia, Policlinico Riuniti di Foggia, Foggia, Italy; 2 Department of Anesthesia, Critical Care and Emergency, Fondazione Istituto di Ricovero e Cura a Carattere Scientifco Ca’ Granda Ospedale Maggiore Policlinico Milan, Milano, Italy; 3 Instiute of Anesthesia and Intensive Care, Padua University Hospital, Padova, Italy; 4 Department of Critical Care Medicine, McGill University, Montreal, Quebec Canada; 5 Anesthesia and Intensive Care Unit, Department of Translational Medicine, University of Ferrara, Ferrara, Italy; 6 Anesthesia and Intensive Care Unit, Department of Medical and Surgical Sciences, “Magna Graecia” University, “Mater Domini” University Hospital, Catanzaro, Italy; 7 Department of Surgical, Oncological and Oral Science (Di.Chir.On.S.), Section of Anaesthesia, Analgesia, Intensive Care and Emergency, Policlinico Paolo Giaccone, University of Palermo, Palermo, Italy; 8 Department of Pathophysiology and Transplantation, University of Milan, Milan, Italy; 9 Department of Medicine – DIMED, University of Padua, Padova, Italy

**Correction: J Anesth Analg Crit Care 2, 28 (2022)**

**https://doi.org/10.1186/s44158-022-00055-6**

Following publication of the original article, the authors identified that the given names and family names of all authors were swapped.

The author group has been updated above and the original article has been corrected.

